# MRI-based radiomics for predicting histology in malignant salivary gland tumors: methodology and “proof of principle”

**DOI:** 10.1038/s41598-024-60200-9

**Published:** 2024-04-30

**Authors:** Zahra Khodabakhshi, Laura Motisi, Andrea Bink, Martina A. Broglie, Niels J. Rupp, Maximilian Fleischmann, Jens von der Grün, Matthias Guckenberger, Stephanie Tanadini-Lang, Panagiotis Balermpas

**Affiliations:** 1 Department of Radiation Oncology, Zurich University Hospital, Zurich, Switzerland; 2 Department of Neuroradadiology, Clinical Neuroscience Center, University Hospital Zurich and University of Zurich, Zurich, Switzerland; 3 Department of Otorhinolaryngology, Zurich University Hospital, Zurich, Switzerland; 4 Department of Pathology and Molecular Pathology, University Hospital Zurich, Zurich, Switzerland; 5 Department of Radiation Oncology, J.W. Goethe University Hospital Frankfurt, Frankfurt, Germany

**Keywords:** Head and neck cancer, Diagnostic markers

## Abstract

Defining the exact histological features of salivary gland malignancies before treatment remains an unsolved problem that compromises the ability to tailor further therapeutic steps individually. Radiomics, a new methodology to extract quantitative information from medical images, could contribute to characterizing the individual cancer phenotype already before treatment in a fast and non-invasive way. Consequently, the standardization and implementation of radiomic analysis in the clinical routine work to predict histology of salivary gland cancer (SGC) could also provide improvements in clinical decision-making. In this study, we aimed to investigate the potential of radiomic features as imaging biomarker to distinguish between high grade and low-grade salivary gland malignancies. We have also investigated the effect of image and feature level harmonization on the performance of radiomic models. For this study, our dual center cohort consisted of 126 patients, with histologically proven SGC, who underwent curative-intent treatment in two tertiary oncology centers. We extracted and analyzed the radiomics features of 120 pre-therapeutic MRI images with gadolinium (T1 sequences), and correlated those with the definitive post-operative histology. In our study the best radiomic model achieved average AUC of 0.66 and balanced accuracy of 0.63. According to the results, there is significant difference between the performance of models based on MRI intensity normalized images + harmonized features and other models (*p* value < 0.05) which indicates that in case of dealing with heterogeneous dataset, applying the harmonization methods is beneficial. Among radiomic features minimum intensity from first order, and gray level-variance from texture category were frequently selected during multivariate analysis which indicate the potential of these features as being used as imaging biomarker. The present bicentric study presents for the first time the feasibility of implementing MR-based, handcrafted radiomics, based on T1 contrast-enhanced sequences and the ComBat harmonization method in an effort to predict the formal grading of salivary gland carcinoma with satisfactory performance.

## Introduction

Salivary gland cancers (SGC) are rare tumors, representing 1–5% of all head and neck cancers and include a wide range of histological features and clinical behaviors, which makes diagnosis and treatment challenging^1–3^. Furthermore, the rarity of these tumors combined with the variable histology lead to a lack of studies that could provide strong recommendations for each individual histological subtype^4^. Taken together, both the rarity and the heterogeneity of these malignancies pose a great challenge that makes general recommendations and randomized trials if not impossible, at least insufficient. There is a general need for a more tailored, individualized treatment and this can only be provided by timely and precise diagnostic approaches.

SGC are generally graded as low grade, high grade or mixed^5^, and to provide a better treatment guidance, it could be important considering, among others, histology and grade^6^. Especially the pretherapeutic histological classification and diagnosis remains a crucial challenge, as it could affect the type and extent of surgery^7^ and consequently affect function and quality of life, or even the need for an additional surgical intervention^8,9^. The consequent risk, if there is a misdiagnosis/undervaluing of the entity of the tumors, can be both an overtreatment and an undertreatment. One main risk is the increased risk for locoregional relapse, if the patient is not operated properly. On the other hand, an extensive surgery in case of low-grade tumors, that could be treated e.g. with partial parotidectomy alone, could lead to detrimental side effects for quality of life, such as facial nerve palsy. Generally, surgical over- and under treatment should be avoided in order to balance between the risk of relapse and the late side effects such as injury of the cranial nerves. Moreover, in the future a detailed radiomic report could augment or even replace molecular pathology in a faster and much less expensive way. Finally, a correct histological diagnosis guides the surgeon regarding extent of neck dissection and reconstruction^8,9^.

Fine needle aspiration biopsy (FNAB) or core needle biopsy (CNB) are strongly endorsed by guidelines^8^, but are often only suitable for distinguishing between malignant and benign tumors, and they are constrained in determining the exact histological grade. That means that these methods alone are always invasive but may only be able to make a limited statement^10^. MR-imaging is nowadays the diagnostic standard for detection and description of the extent and invasion of SGC, but classical radiological features are similar between various malignant subtypes, so they cannot be differentiated by classical imaging alone^11^. Initial diagnosis is mostly performed by cytology (e.g. via fine-needle-aspiration). This procedure is, as said before, invasive and cannot offer a conclusive histology. Moreover, even the grading-discrimination has to be revised in several cases^12^. The overall accuracy to this regard does not exceed 89% even for experienced experts in high-volume centers and can drop down to 31.6% for some more difficult to assess subtypes. A complete histological evaluation is often only possible in the postoperative specimen, as these tumors are in close proximity to vulnerable structures like major vessels and nerves and it is difficult to acquire enough material without additional risks through the biopsy. Finally, as molecular pathology is clearly recommended in order to distinguish between various-often very similar subtypes-^1^, in the future radiomics could allow for a faster and less expensive solution, performed already at the beginning of the diagnostic procedures.

In recent years radiomics has attracted attention of many researchers, thanks to its possibility to convert medical images into data, i.e. make the data behind the images visible (comment: images are data), which can be subsequently analyzed for decision support^13^. Previous studies showed the utility of these quantitative imaging descriptors as prognostic or predictive biomarkers^14–16^.

For head and neck tumors, radiomics is a new research method, which through the characterization of pixel gray level distribution patterns that can be analyzed by machine-learning algorithms, could provide important information about tumor physiology and consequently improve the clinical management of these tumors^17^.

Regarding SGC, there are only very few radiomics studies published so far, based on MRI. Under the combination of the words “radiomics, salivary gland tumors” or “radiomics, salivary glands tumors” or “radiomics, salivary gland tumors, MRI” in Pubmed there are a total of 16 articles. Of these 3 are reviews and 3 being only CT-based. Furthermore, most of these studies tried only to differentiate between malignant and benign parotid gland lesions^18^. This option offers no more information than a FNB or CNAB.

Therefore, the aim of this study was to develop a radiomics method able to distinguish between high- and low-grade salivary gland malignancies. There is a gap of knowledge and difficulties in distinguishing between these two types of tumors regardless of the preoperative diagnostic modality used, with postoperative molecular pathology being the only method providing high validity. MRI is nowadays the diagnostic modality of choice, but there is no real experience published regarding the extraction of MR-radiomic features for such purposes. This is the first MR-radiomic project investigating a larger cohort of patients with such rare tumor entities, with the aim to investigate a new method, leading to an improved and faster diagnosis and consequently a better treatment choice. The presented approach could augment treatment decisions as an additional pre-therapeutic information and may also be used in the future together with other clinicopathological factors as a prognostic biomarker.

## Methods

Here we included patients with histologically confirmed SGC who underwent curative-intent treatment, from 2009 to 2021 in the University Hospitals of Zurich and Frankfurt. Importantly, in the last 20 years, MRI was standard diagnostic modality for all patients with salivary gland tumors in both participating centers. However, the exact timeframe for every center was simply decided based on both availability of patient records and implementation of signed general consent for data use. The patients signed an informed consent, which allowed for collecting pseudonymized health-related data and images and analyze these retrospectively for any future projects. The images were pseudonymized before extraction and there was no possibility to identify the patients based on these. The study, including retrospective data use, was approved by the two local independent ethical committees of the participating centers (approval numbers: No. 30/17, Ethics Committee, University Hospital Frankfurt, Germany and BASEC-No.: 2019-00684 of the cantonal ethics committee of Zurich) and all methods were performed in accordance with the relevant guidelines and regulations.

All the patients included in the study had a diagnostic MRI (a 3 Tesla machine was the standard). Patients without MRI or only one without contrast were excluded from the analysis. The delineation of the tumor volume was performed in the contrast-enhanced T1-weighted-3D-sequences independently by two experienced radiation-oncology specialists. After that, all cases were reviewed together to look for any possible disagreements in order to enhance inter-observer reliability. No major contour deviations could be identified in this peer-review process. All SGC included were categorized according to the 2017 *WHO Classification of Head and Neck Tumors*^19^. Tumors were graded as “low grade” versus “high-grade”. This is a formal grading, as there is no consensus about and it is not in use for every specific histology included here.

**Figure 1:**
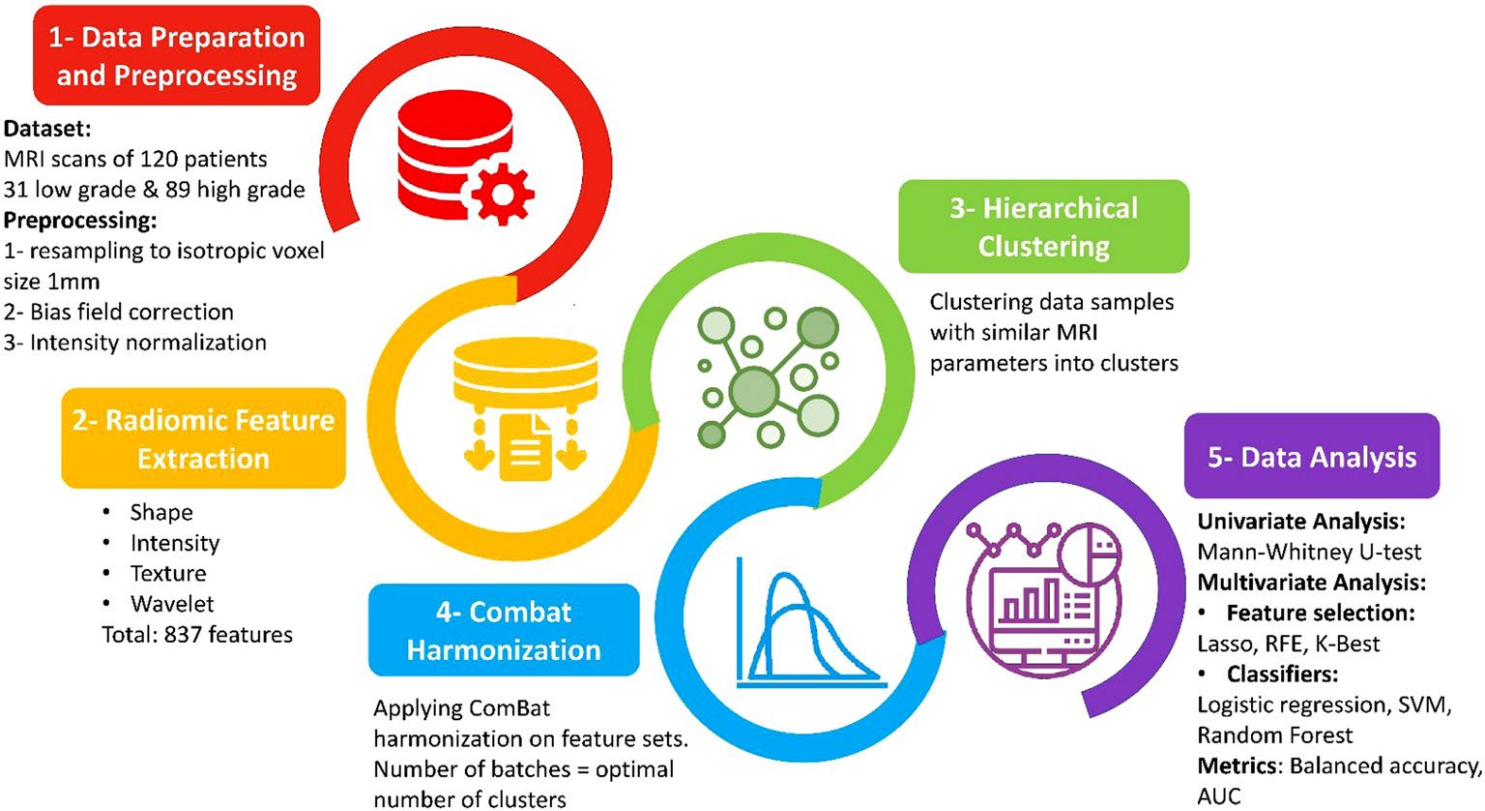
Workflow of the study.

The stepwise approach followed is presented in Fig. 1. The workflow included the following steps: data acquisition, preprocessing, feature extraction, clustering, ComBat harmonization, and univariate and multivariate data analysis.

### Preprocessing

The MRI images were spatially resampled into isotropic voxel size of 1 mm using linear interpolation. N4 bias field correction was applied on the images to correct the spatial intensity inhomogeneities^21^. MR intensity normalization is the crucial step in quantitative MRI analysis due to high sensitivity and variability of MR intensities to different scanners and acquisition parameters^22^. In this study we normalized the data so that the pixel intensity has zero mean and unit variance.

### Feature extraction

The open source Pyradiomics package^23^ was used for radiomic features extraction from the region of interests (ROIs). In total 837 radiomic features extracted including shape features ^14features^, first order intensity-based features (18 features), Gray Level Co-occurrence Matrix (GLCM, 24 features), Gray Level Run Length Matrix (GLRLM, 16 features), Gray Level Size Zone Matrix (GLSZM, 16 features), Gray Level Dependance Matrix (GLDM, 14 features), Neighboring Gary Tone Difference Matrix (NGTDM, 5 features) and wavelet-based features (730 features). Prior to feature extraction the intensities were discretized using a fixed bin number discretization method with 32 bins. All the extracted features are based on the definitions of Standardized Imaging Biomarker Initiatives (IBSI)^19^.

### Unsupervised hierarchical clustering

Samples with similar acquisition parameters were grouped into clusters. Here we relied on unsupervised hierarchical clustering to group similar samples into clusters. This method clusters the data points without prior knowledge based on a similarity matrix using a particular distance metric e.g. Euclidean distance^24^. Silhouette score was used to determine the quality of clustering or the optimal number of clusters. A score close to 1 indicates perfect clustering quality and a negative score suggests miss classification^25^.

### ComBat harmonization

ComBat method is a widely used harmonization method that was primarily used in genomics to remove the batch effects which refers to uncontrollable errors due to non-biological variations, e.g. time and place experimental variation^26^. Several radiomics studies show the sensitivity of radiomic features to the variation in scanner and acquisition parameters (scanner effect). The scanner effect adversely affects the predictive or prognostic performance of radiomic models and limits the use of multicentric dataset to develop generalizable models. Several studies show the effectiveness of ComBat harmonization in removing the scanner effect and increasing radiomic model performance. In this study in addition to MR intensity normalization we applied ComBat harmonization on the extracted features. We considered the number of batches equal to the number of optimal clusters determined in the previous section.

### Machine learning pipeline

**Figure 2:**
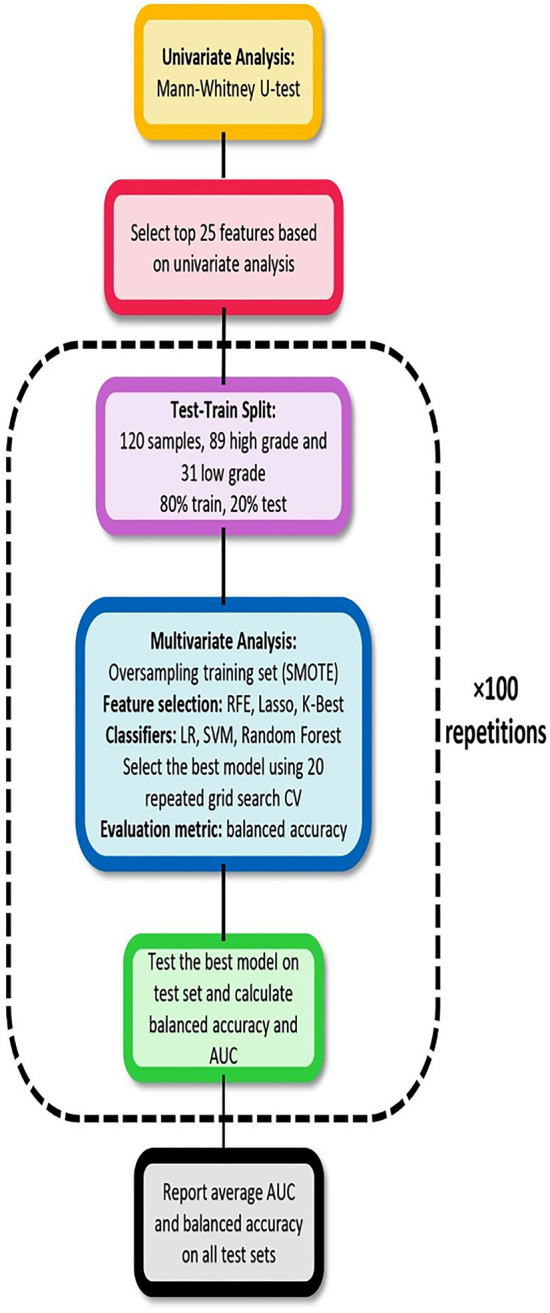
Machine learning pipeline of the study.

In this study we built radiomics models based on 4 different sets of extracted features including: features extracted from non-normalized images, normalized images, non-normalized image + ComBat, and normalized images + ComBat. The overall scheme of the machine learning pipeline is presented in Fig. 2. As the first step we conducted univariate analysis on each feature set. Mann–Whitney U-test was used to calculate AUC and P-values for each radiomics feature. The top 25 features based on their AUC were selected for further analysis in the multivariate section. In order to not limit the conclusions on the results of a specific classifier and feature selection method we used different methods. There are three general categories for feature selection approaches including: filter-based, wrapper, and embedded approaches^27^. In this study, we used K-Best (ANOVA), recursive feature elimination (RFE), and Lasso which are widely used feature selection algorithms belonging to the mentioned categories. For classification we used linear, nonlinear, and boosting algorithms including Logistic Regression (LR), Support Vector Machine (SVM), and Random Forest (RF). The data was split into train and test sets (80% train and 20% test). Model hyperparameter tuning implemented using repeated grid search cross validation and the balanced accuracy was used as evaluation metric. The best model was evaluated on the test set and balanced accuracy and AUC were reported. The procedure of training and testing repeated for 100 iterations for different train and test sets and the average of accuracy and AUC was finally reported. By this methodology we make sure that we have a reliable estimate of model performance and all data samples are included in training and testing. It should be mentioned here that since the data was imbalanced (the low-grade histology was much less frequently respresented) we used synthetic minority oversampling technique (SMOTE) to balance the training set in each iteration. In order to check if intensity normalization and ComBat harmonization have significant effect on radiomics model performance, the Frieman test followed by Conover post hoc analysis were applied on average AUCs and balanced accuracies.

## Results

From the 126 cases examined, we selected a total of 120 patients fulfilling the criteria, 73 male and 47 female, with an age range of 18–92 (Median of 63.2 years). (Table 1. Patient and tumor characteristics).

**Table 1:** Summary of patients’ characteristics.

Characteristics		
Formal grade		low/ high grade
Tumor histology	Acinic cell carcinoma 8	6/2
	Adenocarcinoma NOS (not-otherwise specified) 28	1/27
	Adenoid cystic carcinoma 18	0/18
	Basal cell adenocarcinoma 3	3/0
	Carcinoma ex pleomorphic adenoma 9	0/9
	Carcinoma ex pleomorphic adenoma malignant mixed tumour 1	0/1
	Hyalinizing clear cell carcinoma 1	1/0
	Epithelial myoepithelial carcinoma 5	2/3
	Mucoepidermoid carcinoma (MEC) 16	14/2
	Myoepithelial carcinoma 4	4/0
	Small cell carcinoma 1	0/1
	Squamous cell carcinoma (metastases) 2	0/2
	Squamous cell carcinoma (primary not ruled out) 11	0/11
	Salivary duct carcinoma 11	0/11
	Sebaceous adenocarcinoma 1	0/1
	Spindle cell osteosarcomatoid tumor 1	0/1
Sex (M/F)	73/47	
Age (years)	63.2 (18–92)	
Tumor location	Parotid 109	
	Sublingual 1	
	Submandibular 6	
	Minor salivary glands 4	
Tumor site	Right 57	
	Left 62	
	Middle-line 1	

### Unsupervised clustering

**Figure 3:**
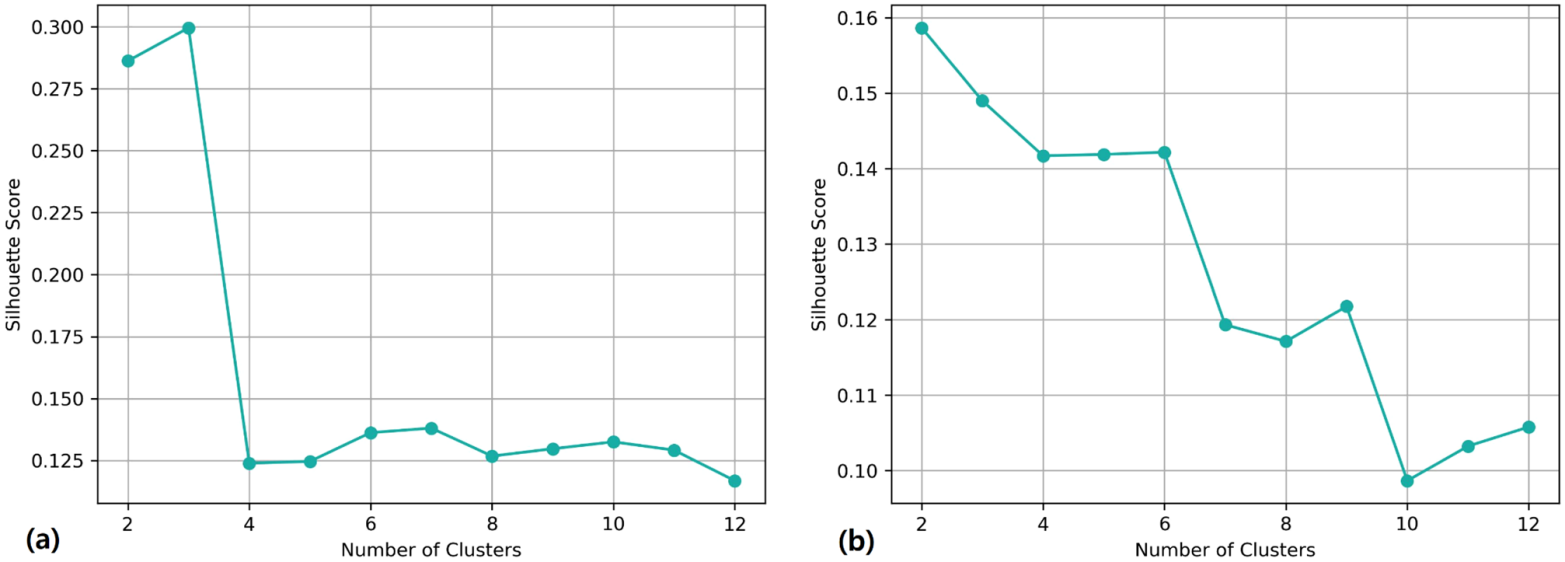
Identifying the optimum number of clusters based on Silhoutte score for (**a**) normalized dataset, (**b**) non-normalized dataset.

The results of unsupervised hierarchical clustering for both normalized and non-normalized MR scans are presented in Fig. 3. Based on the results, increasing the number of clusters resulted in reduction of silhouette score and in both cases the score is lower than 0.5 which indicates that the clusters are overlapped. Based on silhouette score the optimum number of clusters for non-normalized and normalized datasets are 2 and 3 respectively. However, for normalized dataset we also consider only two clusters, since in order to successfully remove the scanner effect using ComBat harmonization at between 20 and 30 representative data samples in each cluster are required^26^ but the third cluster in normalized dataset only has two samples. The clusters are represented as dendrograms in Supplementary Fig. 1.

### Univariate analysis

Mann-Whithney U-Test was applied on normalized and non-normalized data sets before and after ComBat harmonization. The AUCs and *p* values for top 10 features based on the AUCs are represented in Table 2.

**Table 2:** AUC and *p* values of top ten radiomic features for normalized and non-normalized images before and after applying ComBat harmonization.

Feature	AUC	*P* value
**Normalized**		
wavelet-HLH_glrlm_LongRunLowGrayLevel Emphasis	0.650	0.012
wavelet-HLH_gldm_Dependence Variance	0.648	0.014
wavelet-HLH_glcm_Maximum Probability	0.644	0.016
wavelet-HHL_girlm_LongRunLowGrayLevel Emphasis	0.644	0.017
Normalized wavelet-HLH_gldm_Large DependenceLowGrayLevelEmphasis	0.644	0.017
wavelet-LHH_firstorder_Kurtosis	0.643	0.017
wavelet-LLH_glcm_Imc2	0.643	0.017
wavelet-LLH_glcm_MCC	0.642	0.018
wavelet-HLH_firstorder_Skewness	0.642	0.018
wavelet-LLH_gldm_Large DependenceHigh GrayLevel Emphasis	0.639	0.020
**Normalized + Combat**		
original_glszm_GrayLevelVariance	0.655	0.010
wavelet.HLH_ngtdm_Strength	0.649	0.013
ComBat wavelet.LLH_ngtdm_Strength	0.648	0.014
wavelet.LLH_glcm_Imc2	0.647	0.014
wavelet.HLH firstorder Skewness	0.646	0.015
wavelet.HHL_glrlm_LongRunLowGrayLevel Emphasis	0.646	0.015
Normalized wavelet.HLH_gldm_DependenceVariance	0.641	0.019
wavelet.HHL_glszm_LowGrayLevelZoneEmphasis	0.638	0.022
wavelet.LLH_glcm_MCC	0.636	0.023
wavelet.HLL_ngtdm_Complexity	0.636	0.024
**Non-Normalized**		
wavelet-HLH_firstorder_Minimum	0.668	0.005
wavelet-HLH_firstorder_Skewness	0.647	0.014
wavelet-HLH_glrlm_LongRunLowGrayLevelEmphasis	0.646	0.015
wavelet-LLH_glcm_Imc2	0.646	0.015
wavelet-HHH firstorder Minimum Normalized	0.645	0.016
wavelet-LLH_glcm_MCC	0.642	0.018
No-wavelet-LHH firstorder Kurtosis	0.640	0.020
wavelet-HLH_gldm_Dependence Variance	0.640	0.020
wavelet-LHH_firstorder_Minimum	0.639	0.020
wavelet-HLH_gldm_Large Dependence LowGrayLevelEmphasis	0.638	0.022
**Non-Normalized + Combat**		
wavelet.HLH firstorder Minimum	0.660	0.008
original_glszm_GrayLevel Variance	0.650	0.012
ComBat wavelet.HHH firstorder Minimum	0.633	0.027
+original_shape_Sphericity	0.632	0.028
wavelet.HHH_glszm_GrayLevel Variance	0.632	0.029
wavelet.LLH_glcm_Imc2	0.629	0.031
Normalized wavelet.HLH firstorder Skewness	0.628	0.034
-wavelet.LHL glrlm_ShortRunHighGrayLevelEmphasis	0.626	0.036
No wavelet.HHL_glszm_LowGrayLevelZone Emphasis	0.624	0.040
wavelet.LHH_glszm_SmallAreaLowGrayLevelEmphasis	0.620	0.046

According to the table, the highest AUCs are 0.650, 0.655, 0.668, 0.660 which belong to wavelet-HLH_glrlm_LongRunLowGrayLevelEmphasis, original_glszm_GrayLevel_variance, wavelet-HLH_firstorder_Minimum respectively. Based on the table the top 10 features are mostly from the wavelet category. Sphericity is the only feature from the shape feature category with AUC and P-value of 0.63 and 0.02 respectively.

### Multivariate analysis

**Figure 4:**
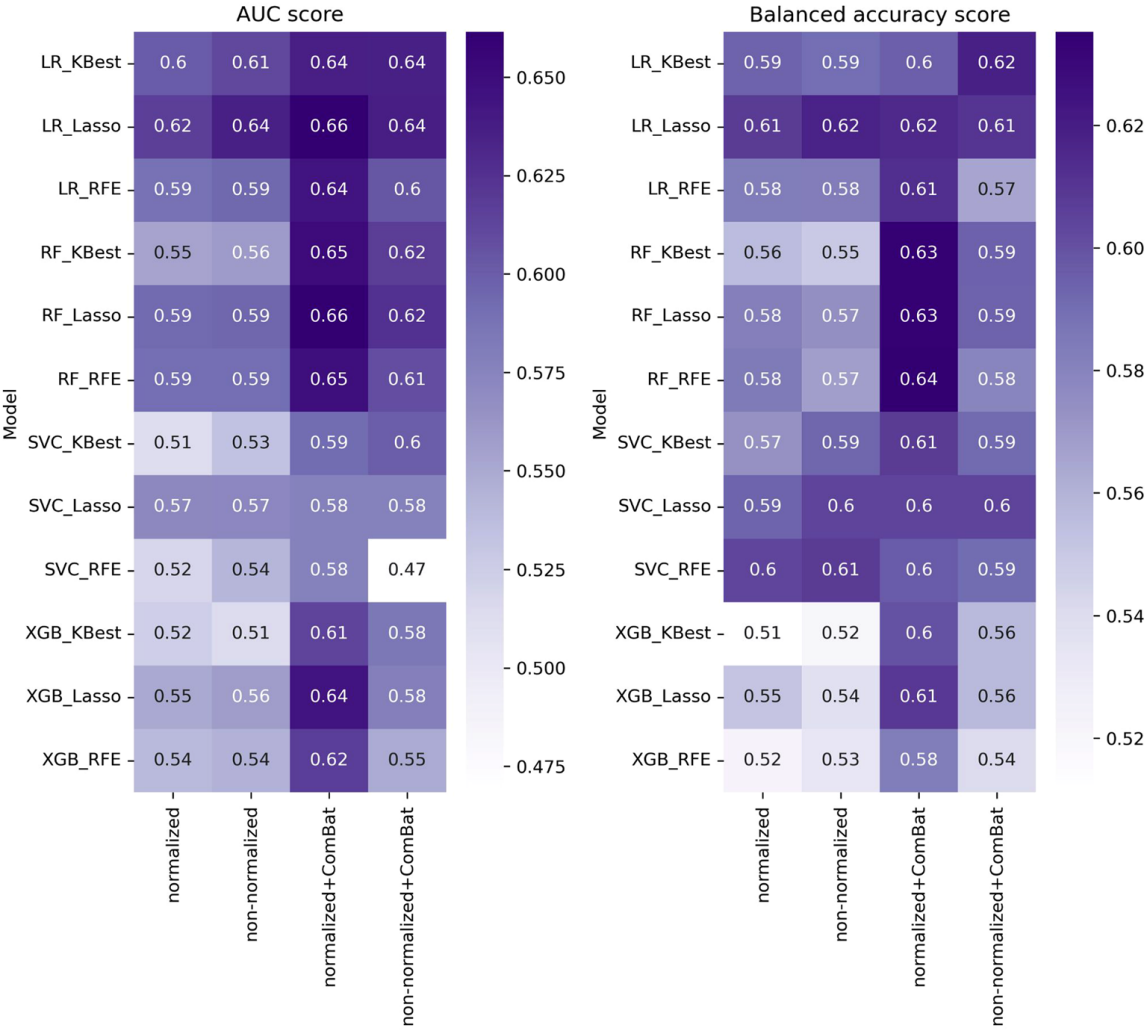
Average AUC and balanced accuracy for different datasets and different combination of classifiers and feature selectors.

The results of multivariate analysis in terms of average AUCs and balanced accuracies are represented in Fig. 4. According to the results, the random forest classifier achieved the highest AUC and balanced accuracy of 0.66 and 0.64 respectively. Between the four datasets used in our analysis, intensity normalized images in combination with ComBat feature harmonization could increase the performance of radiomic models.

**Figure 5:**
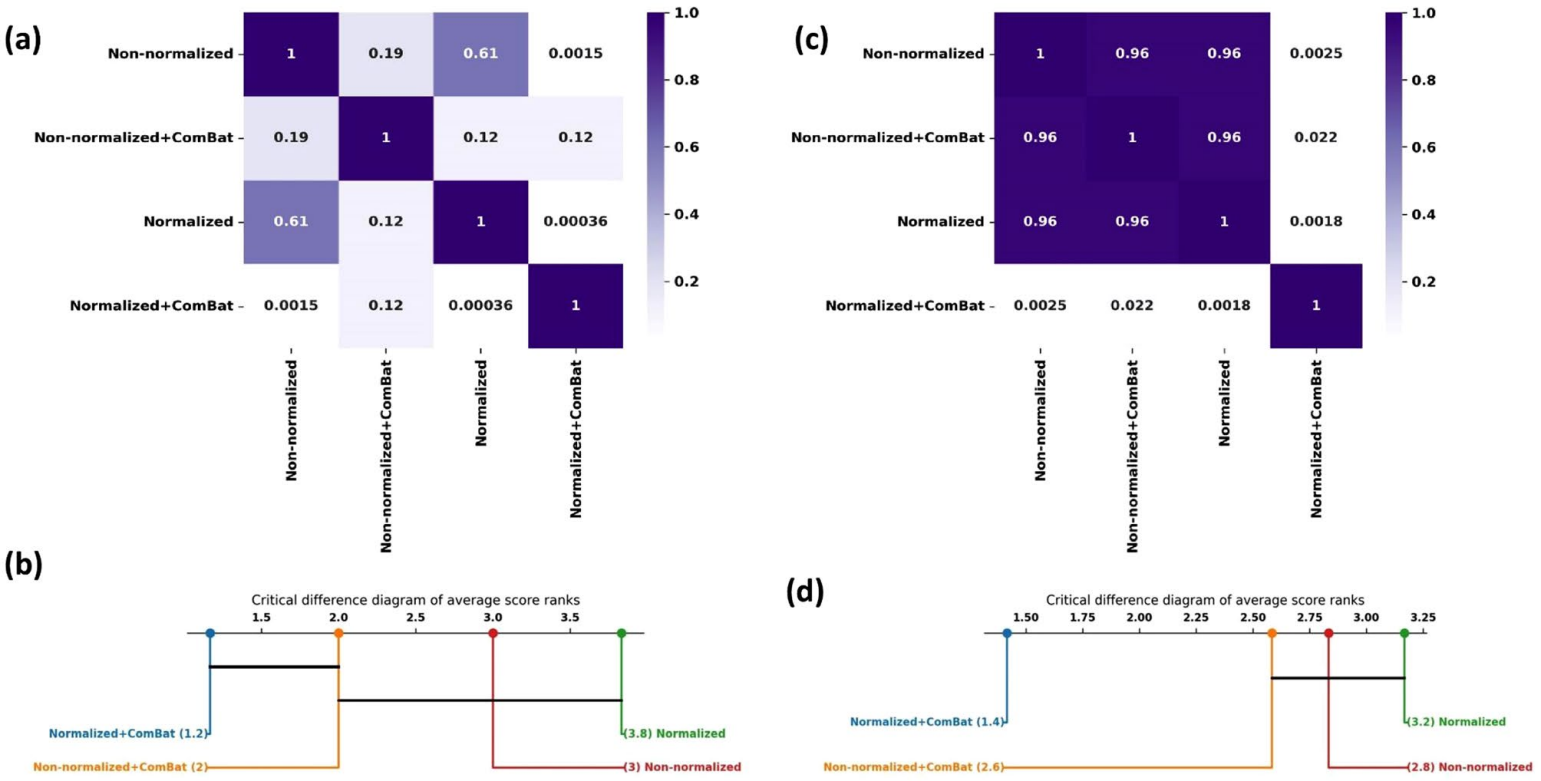
Heatmap of *P* values and critical difference plot of radiomics model performance in terms of AUC and balanced accuracy based on different datasets. (**a**) Heatmap of AUCs *p* values, (**b**) critical difference plot based on AUCs. (**c**) Heatmap based on balanced accuracy *p* values. (**d**) critical difference plot based on average balanced accuracy.

The Friedman test was applied on both AUCs and balanced accuracies and the corresponding p-values were 0.00002 and 0.005 which indicates that there is significant difference between the performance of radiomics models based on different datasets. The p-values based on Conover post hoc analysis and critical difference plots are represented in Fig. 5. In the critical difference plot the most left vertical line represents the dataset which results in better performance and the right line represents the worse one. The horizontal lines connect cases with similar performance. Based on Fig. 5b, d intensity normalized images followed by ComBat harmonization has the best rank in terms of AUC and balanced accuracy. Also, non-normalized image, non-normalized + ComBat and normalized images resulted in similar performance.

**Figure 6:**
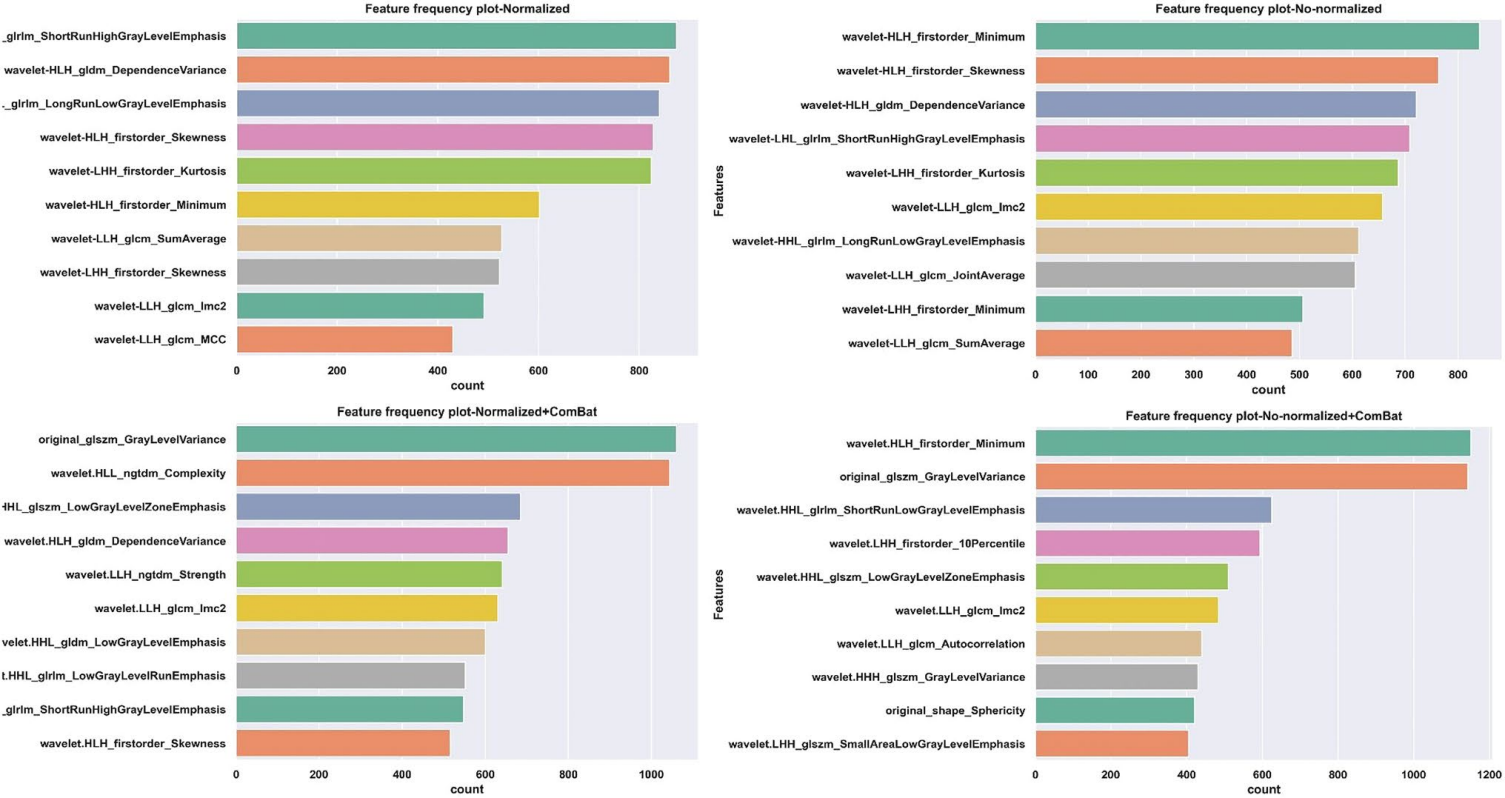
Count plot of high frequently selected features for extracted features based on (**a**) Normalized (**b**) Normalized + ComBat (**c**) No-normalized (**d**) No-normalized + ComBat.

The count plots of top 10 frequently selected features during training procedures are represented in Fig. 6. For both no-normalized and no-normalized image + ComBat wavelet-HLH-original—first order-minimum has been frequently selected during the training process and achieved the first rank. For normalized and normalized + Combat the first rank features based on the countplot are wavelet-LHL-glrlm-ShortRunHighGrayLevelEmphasis and original-glszm-GrayLevelVariance.

## Discussion

In the present pilot study, the primary objective was to investigate the potential of MRI-radiomic based models for predicting high-grade versus low-grade histology of untreated salivary gland cancer. Secondly, the usage of harmonization in improving the performance of such models was demonstrated, as there was a significant difference between the performance of models based on MRI intensity normalized images and harmonized features and other models. The best radiomic model achieved average AUC of 0.66 and balanced accuracy of 0.63, which can be deemed satisfactory when the limitations of the study are being considered. However, there is surely room for improvement. This is to the best of our knowledge one of the first efforts to characterize the histology of SGC by the means of radiomics, the largest cohort reported so far and the first one introducing the specific methods described above. This methodology could be implemented in a larger cohort to provide more valid conclusions and even a precise prediction of the exact histopathological subtype.

The correct pre-therapeutic classification of salivary gland neoplasms remains of crucial interest for pre-operative decision making. The extent of surgery, i.e. partial or total parotidectomy, sacrificing the facial nerve (at least indirectly, based on aggressiveness/ infiltration) and the decision for or against a neck dissection or the probability of postoperative radiotherapy strongly depends on the histopathological findings ^28,29^. Unfortunately, this information can only be obtained after initial surgery, as open biopsies should be avoided. Although nowadays FNAB and CNB are considered sensitive and specific enough to differentiate between benign and malignant neoplasms (57–86% and 87–100% respectively) they may provide some hints regarding the exact histology or the grading, which often have to be revised after postoperative pathological examination of the tissue, in particularly in centers without access to molecular pathology. Interestingly, even post hoc revisions of the histology or the malignancy of the tumors seem to be a not so rare phenomenon^30^, underlining the unmet need for additional diagnostic methods and work up. Finally, both of the methods reported above are invasive procedures, still harboring the risk of post-interventional complications of any grade, in at least 14% of the patients^31^ with CNB being associated with a slightly increased rate of sequela^28^.

Karimian et al. investigated the utilization of FDG-PET-CT to characterize high-grade malignancies in 45 patients and could demonstrate that the tracer uptake positively correlated with the grading^29^. In that study no machine learning model was trained or tested on data, but the correlation between SUV and tumor grade was investigated and the dataset was relatively small and also imbalanced (41 cases including 24 high grade, 9 low grade and 8 intermediate grade). MR-radiomics have been utilized before to address functionality of salivary glands and more precisely to predict xerostomia based on post-treatment alterations^32,33^. In recent years, first efforts to implement distinct imaging features for improving histology prediction have been made. Zheng et al. developed a nomogram in a total of 115 patients after analyzing 17 radiomic MR-features in order to differentiate benign from malignant parotid lesions. In a newer study, topology-based properties of two-dimensional MR-images of 39 patients were evaluated to predict malignancy grade in parotid gland cancer^34^. Although the cohort was very small and only limited features were examined, this study was one of the first to implement radiomics for this exact purpose. The same group reports also of another approach, namely six conventional machine-learning and five deep learning (DL) algorithms ^35^. Despite the small sample size both studies provided promising results regarding performance, with higher AUC, ACC and sensitivity as in the present study. One possible explanation is that the very small dataset is totally balanced and therefore it was less challenging to provide such results. Second, the variation in the MRI scans was less, as the study was unicentric and with fewer cases and both T1-weighted and T2-weighted sequences were used for all patients. Similar to our findings, features associated with tumor heterogeneity seem to play an important role for grading classification. Moreover, this has been previously the case also in radiological investigations, where heterogeneous enhancement and cystic and necrotic parts are generally correlated with high-grade features^11^. Almost all of the significant features in the present study were wavelet features and could be generally explained by the heterogeneity observed by radiologists. Intriguingly, also the most important non-wavelet feature observed here, namely “sphericity” might be explained by the ill-defined borders observed in highly malignant lesions as described before^11,36^.

In contrast to other studies, the present dataset was highly heterogeneous, both in terms of included cases and in terms of acquired imaging. Therefore, we also investigated the effect of MRI intensity normalization and feature harmonization in reducing the variability in the dataset and better performance of the radiomic model. This becomes even more important in the case of salivary gland tumors, one of the most heterogeneous tumor entities, comprising more than 20 different histological subtypes. Based on our results, intensity normalized images in combination with ComBat harmonization significantly improved the performance of the current radiomic model, specifically in comparison to models based on only normalized images without ComBat harmonization, the average improvement was 6.3% and 4% for AUC and balanced accuracy respectively. Interestingly, almost all of the important features resulting in high AUCs were associated with wavelet parameters, with only the feature “sphericity” resulting in comparable results. According to the literature, malignant parotid tumors are linked with ill-defined and infiltrative margin, low and inhomogeneous signal intensity on T1 contrast MR scans^37^. In our study, minimum intensity and gray level variance were reported as high rank and frequently selected features. According to the results of univariate analysis, for non-normalized images the minimum from the first order feature category repeatedly appears in the table as a feature with high AUC. This feature represents the minimum intensity inside the tumor region. In the existing literature, malignant parotid tumors are linked with low intensity on T1 MRI scans^37^. Since some of the low grade malignant SGC tumors can also represent benign tumor characteristics it can be inferred that high grade SGC are linked with lower signal intensity in tumor region and low grade are linked with higher signal intensity. Between the morphological features, sphericity also has a relatively high AUC of 0.63. This feature quantifies how the tumor shape is close to a perfect sphere like volume^38^. Some studies indicate that malignant parotid tumors are characterized with an ill-defined margin. It can be inferred that low grade SGC may represent a more sphere-like shape than high grade lesions. From texture feature category, dependence-variance, gray level variance, low and high gray level emphasis are between top features. All these features quantify the heterogeneity of gray levels or intensities inside the ROI. It has been already reported, that the malignancy of parotid tumors could be linked with intensity heterogeneity within the tumor region^37^.

All of the extracted features in our study were defined in compliance with IBSI. Moreover, the preprocessing was based on IBSI recommendations for MRI radiomic studies. In overall, intensity discretization is a fundamental pre-processing step in a radiomic workflow in order to suppress the existing noise in the image and make the subsequent calculation of texture features tractable. There are two methods of intensity discretization including fixed bin size and fixed bin number (FBN). According to IBSI documentation the intensity discretization method is modality dependent. Since the FBN method applies a normalization effect on the image, it would be beneficial to use it on a raw MRI dataset. Moreover, FBN adjusts the contrast between the images^38^. According to the radiomic features definitions provided by Image Biomarker Standardized Initiative (IBSI)^38^, minimum intensity is the minimum intensity of voxels included in the region of interest (here tumor region), and gray level-variance (specifically GLSZM gray level variance in our study) “estimates the variance in zone (linked voxels with identical intensities) counts over the gray levels”^38^. Minimum intensity correlates with necrosis in tumor region and gray level variance is related to the tumor heterogeneity.

In our study, we were dealing with a highly heterogeneous MRI dataset and the scans were acquired with more than 20 different MRI acquisition parameters. In order to harmonize the data (using ComBat method) we needed to group the similar scans into clusters. To do so we implemented unsupervised hierarchical clustering. Different clustering approaches are proposed in the literature and the most popular methods are: unsupervised hierarchical clustering, K-means, and Expectation Maximization (EM)^39^. In agglomerative clustering (the method implemented here), each data sample is considered as a cluster initially and then each pair of similar clusters are merged together. The procedure continues and at the end there are only two clusters to merge. In contrast to K-mean and EM clustering methods, in hierarchical clustering there is no need to predefine the number of clusters and it should be noted that the former algorithms strongly depend on the initial assignment of number of clusters. If there is no clue about the approximate number of clusters, the algorithm should run for several random numbers of clusters^40^. Since we had several different MRI acquisition parameters, we decided to use hierarchical clustering to select the optimum number of clusters (similar scans) automatically.

There are lots of studies which show the effectiveness of ComBat harmonization in removing the center effect in radiomic studies with different modalities including PET, CT and MRI^41–43^. Orlhac et.al^44^ first used the ComBat harmonization to harmonize the features extracted from healthy liver tissue and breast tumors based on FDG-PET scans acquired in two different centers. Based on their results the SUV and features extracted from healthy liver tissue were different in two centers but after applying ComBat harmonization the distribution of features overlapped greatly. Also, the features distributions of triple negative breast cancer patients were more comparable after ComBat Harmonization^44^. In another study Mahon et al.^45^ investigated the effect of ComBat harmonization on extracted features from the CT scans of lung phantom and human subjects. The scans were acquired using 32 different chest imaging and 6 different thorax imaging protocols for phantom and human subjects respectively. According to their results, ComBat harmonization could reduce the percentage of features with significantly different distribution to 0–2% and 0–19% for phantom and human subjects respectively^45^. Another study investigated the effectiveness of ComBat harmonization on performance of a classification radiomics model based on a heterogeneous MRI dataset. Considering all feature categories, ComBat harmonization could increase the model accuracy significantly. In that study two different variants of ComBat were implemented and both improved the results in comparison to unharmonized data^46^.

The main objective of our study was to investigate the power of radiomic features to distinguish between high grade and low grade SGC and we did not consider the significance of other variables. In a study by Baba et al.^47^ the impact of age and gender was investigated. According to their results, patients with high grade parotid tumors were significantly older than patients with low grade tumors. Moreover, patients with high grade tumors were mostly male. Age and gender could be considered as potential additional variables to be integrated together with radiomics in a future model in order to predict high grade and low-grade parotid tumors.

There are limitations to this study. First, the still limited number of patients included, as well as the imbalance between low- and high-grade cases. Unfortunately, this represents the epidemiological “real-world” situation of these rare tumors. However, it should be mentioned that there were also other limitations which hindered the model to reach higher performance. The main challenge was data heterogeneity. In this study the MRI scans were acquired with more than 20 different acquisition parameters. Considering large variation in intensity range of the MRI images due to variation of scanner and protocol made our study more challenging. Please note that even scans from the same patient with the same scanner and same parameters but at different time points do not have the same intensity range^48^. However, we implemented harmonization in both image and feature domain to address this problem. In the real word domain most of the datasets are imbalanced and one class is less represented than the other class. This was also the case in our study. With an imbalanced dataset, the performance of the classification algorithms tends to be biased toward the majority class since the existing algorithms assume equal distribution of both classes and tend to minimize the overall error in which the minority class contributes less^49^. There are some approaches to address this issue, including undersampling of the majority class, oversampling of the minority class, and using cost sensitive algorithms^50^. In our study we used the synthetic minority oversampling technique to oversample the minority class. Second the different MRI-vendors and platforms used for image acquisition, as well as possible differences in protocols, e.g. regarding slice thickness and contrastat agent volume used in each case. The ComBat harmonization method can account for this variability, but it is also limited by inability to harmonize multimodal distributions, unknown and multiple imaging parameters^51^. Last but not least, although the high percentage of NOS-adenocarcinomas (“NOS adenocarcinomas” or salivary gland adenocarcinoma not otherwise specified is according to the WHO classification a tumor without the histological features characteristic of other cancer types). included here corresponds to larger series published previously, it seems a bit unusual under today`s point of view. This can be attributed to the long inclusion-interval for the cases presented here, as elaborate molecular analyses were not established in the past. All of the above limitations may affect the generalizability of the results. The conclusions drawn here should firstly be validated in an external cohort and then the method integrated in a prospective trial before being suitable for clinical routine.

Nevertheless, the innovative character of the idea, as it is not only one of the first, but the largest and only bicentric study examining the topic and the encouraging performances observed could serve as a paradigm for future larger cohorts trying to answer the unmet clinical need of pre-therapeutic histological prediction in SGC.

## Conclusion

The present bicentric study presents for the first time the feasibility of implementing MR-based, handcrafted radiomics, based on contrast-enhanced T1-weighted- sequences and the ComBat harmonization method to predict grading of salivary gland carcinoma with satisfactory performance. Further research and larger cohorts are needed to validate these observations and make additional and more specific predictions.

### Supplementary Information

Supplementary Figure 1.

## Data Availability

The datasets generated during and/or analyzed during the current study are available from the corresponding author on reasonable request.
