# Supplementary material for: MRI-based radiomics for predicting histology in malignant salivary gland tumors: methodology and “proof of principle”
Source: Sci Rep. 2024 Apr 30;14:9945. doi: 10.1038/s41598-024-60200-9 (PMC11061101; doi:10.1038/s41598-024-60200-9)
Supplement: Supplementary file 1 — Supplementary Figure 1. [file 41598_2024_60200_MOESM1_ESM.docx]

***Supplementary materials:***

-
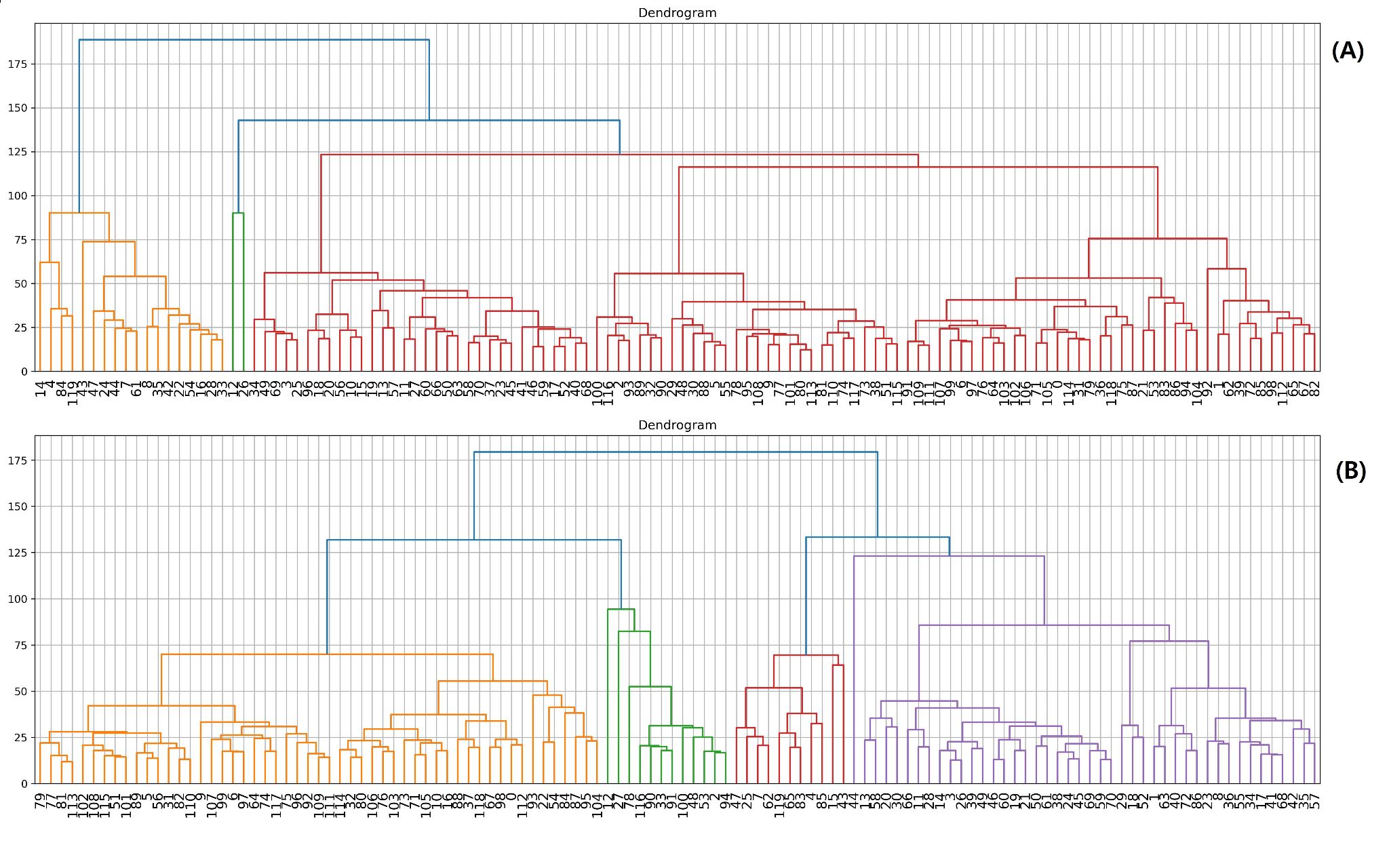

- ***Supplementary Figure 1.*** *Dendrogram based on unsupervised hierarchical clustering for* ***A)*** *features extracted from normalized images and* ***B)*** *features extracted from images without intensity normalization*
